# Regional Fascial Plane Blocks and Early Postoperative Complications in Reduction Mammaplasty: A Retrospective Cohort Study

**DOI:** 10.1007/s00266-026-06068-8

**Published:** 2026-06-15

**Authors:** Handan Derebaşınlıoğlu, Onur Avcı, Murat Çelik, Mesut Dağdeviren

**Affiliations:** 1 Department of Plastic, Reconstructive and Aesthetic Surgery, Sivas Cumhuriyet University, 58070 Sivas, Turkey; 2 Department of Anesthesiology and Reanimation, Sivas Cumhuriyet University, Sivas, Turkey

**Keywords:** Reduction mammaplasty, Regional fascial plane block, PECS II, Serratus anterior plane block (SAPB), Hematoma, Surgical complications

## Abstract

**Background:**

Regional fascial plane blocks are widely used in breast surgery for postoperative analgesia. While analgesic benefits are established, their impact on early complications after reduction mammaplasty remains unclear.

**Objective:**

To evaluate the association between regional fascial plane block use and early postoperative complications following superior pedicle reduction mammaplasty.

**Methods:**

This retrospective cohort study included 144 consecutive patients undergoing primary bilateral superior pedicle reduction mammaplasty at a single tertiary center. Patients were grouped as block (n=64) or no-block (n=80). All blocks were performed postoperatively at the end of surgery. The primary outcome was a 30-day composite complication, defined as hematoma requiring reoperation, wound dehiscence, or surgical site infection. Multivariable logistic regression assessed the independent association between block use and complications.

**Results:**

Overall, 55 patients (38.2%) developed at least one early postoperative complication. The composite complication rate was higher in the block group than in the no-block group (46.9% vs. 31.3%); however, this difference did not reach statistical significance (p=0.060). In multivariable analysis, block use was not independently associated with complications, whereas total excision weight was the only significant predictor. Hematomas occurred only in the block group (7.8% vs. 0%, p=0.016).

**Conclusions:**

Regional fascial plane block use was not independently associated with overall postoperative morbidity after adjustment. Although hematomas were observed only in the block group, the low event rate and non-randomized design preclude causal inference. However, a relevant increase in overall complication risk cannot be ruled out; therefore, further prospective studies are needed to clarify this relationship.

**Level of Evidence III:**

This journal requires that authors assign a level of evidence to each article. For a full description of these Evidence-Based Medicine ratings, please refer to the Table of Contents or the online Instructions to Authors www.springer.com/00266.

## Introduction

Reduction mammoplasty is one of the most commonly performed procedures in aesthetic and reconstructive breast surgery and is generally associated with high patient satisfaction and functional improvement [1]. Despite its favorable safety profile, early postoperative complications—particularly hematoma, wound dehiscence, and infection—remain clinically relevant [2]. These complications may require reintervention, prolong recovery, and affect aesthetic outcomes, which are central to patient expectations in this setting[3, 4].

Regional fascial plane blocks, including serratus anterior plane block (SAPB) [5], pectoral nerve block type II (PECS II) [6], and serratus posterior superior intercostal plane block (SPSIP) [7], are increasingly used in breast surgery as part of multimodal analgesia strategies [8]. Their analgesic and opioid-sparing effects are well established; however, their impact on surgical safety in reduction mammoplasty remains unclear [9, 10].

Given the anatomical proximity of these injection planes to thoracic wall vascular structures, a potential effect on postoperative bleeding has been suggested [11]. Although bleeding-related complications such as hematoma have been described following thoracic interfascial plane blocks [12], their impact on postoperative complication profiles in reduction mammoplasty remains poorly defined.

**Fig. 1:**
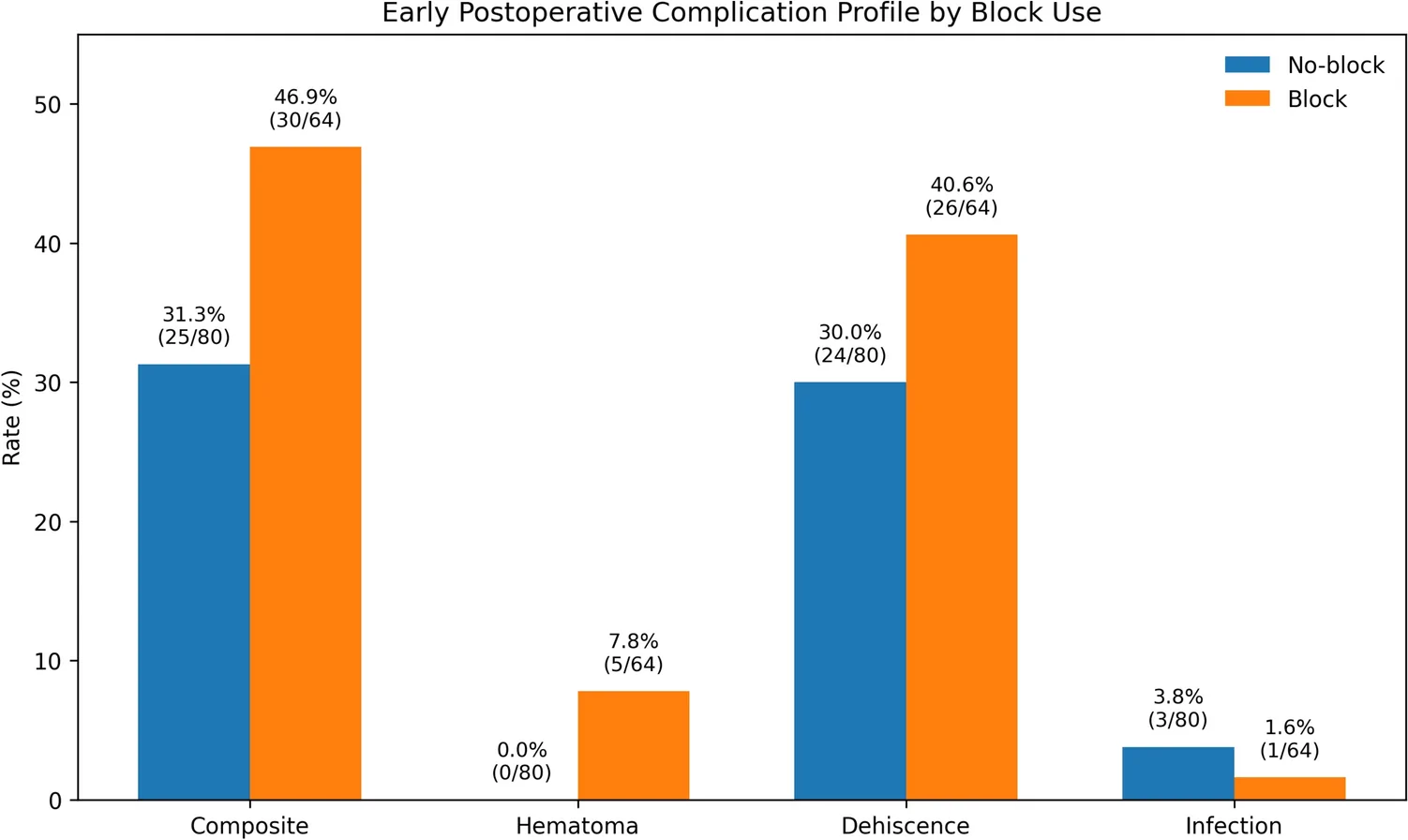
Early postoperative complications by block use. Comparison of early postoperative complications between patients with and without regional fascial plane blocks. Composite complications, hematoma, wound dehiscence, and infection are shown. Values are presented as % (n/N). Higher rates of composite complications, hematoma, and wound dehiscence were observed in the block group, while infection rates were similar between groups

In aesthetic breast surgery, even minor complications can negatively affect patient satisfaction, which represents a key outcome measure following reduction mammaplasty [13, 14]. Evaluating overall complication patterns, rather than isolated outcomes, may therefore provide a more clinically meaningful assessment of surgical safety. In this context, we aimed to evaluate the association between regional fascial plane block use and early postoperative complications following reduction mammoplasty, using a composite outcome to reflect overall surgical safety (Fig. 1).

## Materials and Methods

This retrospective cohort study was conducted at a single tertiary referral center after institutional ethics committee approval. Consecutive adult female patients who underwent primary, bilateral, non-oncologic superior pedicle reduction mammoplasty between June 1, 2021, and December 1, 2025 were identified from electronic medical records. All eligible consecutive patients during the study period were included. Revision procedures, oncologic cases, and procedures involving concurrent breast operations were excluded. One patient with immune thrombocytopenic purpura was excluded because of known coagulopathy (Table 1).

**Table 1:** Baseline characteristics of the study population

Variable	No-block (n=80)	Block (n=64)	p-value
Age (years)	39.05 ± 12.68	38.02 ± 12.59	0.626
BMI (kg/m^2^)	28.27 ± 4.71	28.11 ± 3.12	0.839
Right excision weight (g)	827.1 ± 340.4	672.1 ± 332.9	0.007*
Left excision weight (g)	797.3 ± 335.4	659.0 ± 338.4	0.015*
Preop right SNN (cm)	30.65 ± 3.71	29.44 ± 2.64	0.031*
Preop left SNN (cm)	30.38 ± 3.53	29.48 ± 2.67	0.139
Postop SNN (cm)	19.64 ± 0.88	20.18 ± 0.59	0.001*

Block subtype distribution (block group only): SAPB 15 (23.4%); PECS II 33 (51.6%); SPSIP 16 (25.0%).

Values are presented as mean ± Standard Deviation (SD) or n (%). Comparisons were performed using appropriate parametric or nonparametric tests. BMI, body mass index; SNN, sternal notch-to-nipple distance; SPSIP, serratus posterior superior intercostal plane; PECS II, pectoral nerve block type II; SAPB, serratus anterior plane block; (p < 0.05)

*Statistically significant (p < 0.05)

**Fig. 2:**
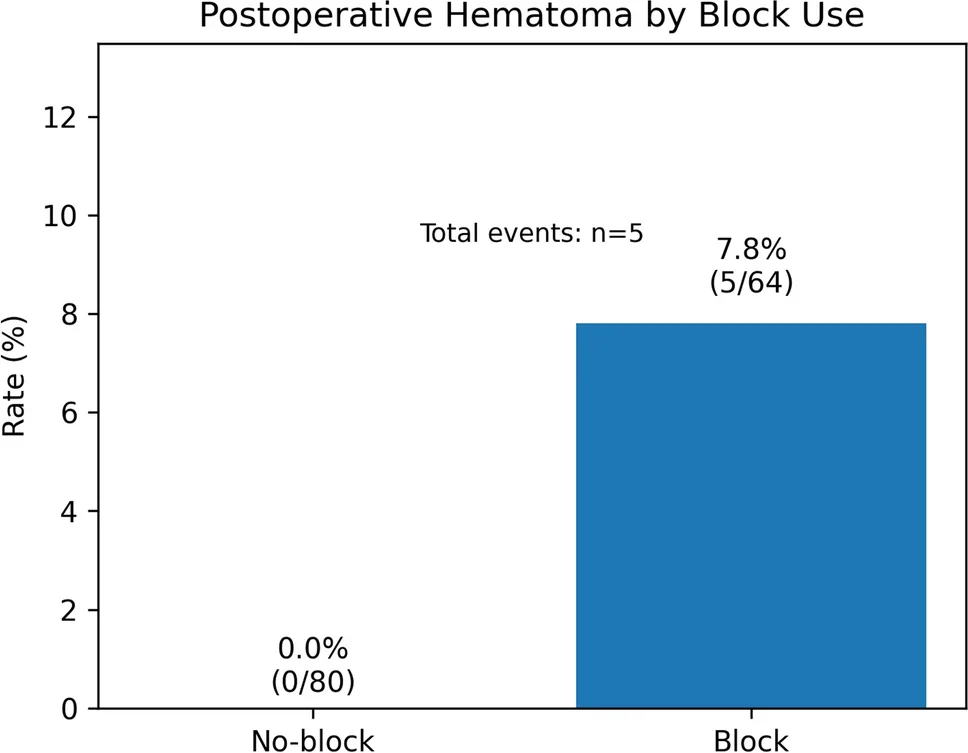
Hematoma rates according to block use. Hematoma occurred only in patients who received a regional block (7.8%, 5/64), with no cases in the no-block group (0/80)

Cases with missing data in variables required for the main analyses were also excluded, and no imputation was performed. Elective reduction mammoplasty was not routinely performed in patients with major comorbidities at our institution; accordingly, the study cohort did not include patients with active smoking, diabetes mellitus, or hypertension (Fig. 2).

Postoperative outcomes were assessed within 30 days using electronic medical records and routine outpatient follow-up. Evaluations or interventions performed at outside institutions were not systematically available; therefore, follow-up completeness could not be formally quantified. The study was conducted and reported in accordance with the STROBE guidelines [15].

### Surgical and Perioperative Standardization

All procedures were performed by a single surgeon using a consistent superior pedicle reduction mammoplasty technique. Perioperative anesthetic management was provided by the same anesthesiologist. Closed-suction drains were routinely placed and removed once output decreased below 20 cc/day according to institutional practice (Table 2).

**Table 2:** Postoperative outcomes according to block use

Outcome	No-block (n=80)	Block (n=64)	p-value
Composite complication	25 (31.3%)	30 (46.9%)	0.060
Hematoma	0 (0%)	5 (7.8%)	0.016*
Wound dehiscence	24 (30.0%)	26 (40.6%)	0.183
Infection	3 (3.8%)	1 (1.6%)	0.629
Drain duration (days)	4.05 ± 1.24	3.97 ± 1.47	0.516
Length of stay (days)	4.16 ± 1.21	4.17 ± 1.22	0.975

Values are presented as n (%) or mean ± SD. Comparisons were performed using appropriate parametric or nonparametric tests. (p <0.05.)

*Statistically significant (p < 0.05)

### Regional Block Protocol

Patients were categorized according to regional fascial plane block use. For the main analyses, block exposure was treated as a binary variable (block vs. no block). Among patients who received a block, subtypes were classified as SAPB, PECS II, or SPSIP blocks. Block selection reflected routine clinical practice and was based on the anesthesiologist’s preference rather than a predefined protocol. Surgeons were not involved in this decision.

All blocks were performed postoperatively under ultrasound guidance using a standardized sterile technique. In all block groups, 30 mL of 0.25% bupivacaine without epinephrine was administered bilaterally. All blocks were performed at the end of the surgical procedure, before the patient was awakened. Analyses according to block subtype were considered exploratory.

### Data Collection

Available demographic and perioperative variables were obtained from electronic medical records, including age, body mass index, preoperative and postoperative sternal notch-to-nipple distances, resection weight for each breast, drain duration, length of hospital stay, and postoperative complications. For regression analyses, total excision weight was calculated as the sum of right and left breast resection weights and used as a surrogate for surgical extent. Nipple transposition distance was defined as the difference between preoperative and postoperative sternal notch-to-nipple measurements (Table 3).

**Table 3:** Multivariable logistic regression for composite complications

Variable	OR	95% CI	p-value
Block use (yes vs. no)	2.45	0.97–6.17	0.058
Total excision weight (per gram increase)	1.001	1.000–1.002	0.016*
Age (years)	1.00	0.96–1.04	0.941
BMI (kg/m^2^)	0.99	0.87–1.13	0.882
Nipple transposition distance (cm)	1.20	0.98–1.47	0.084

Odds ratios (ORs) are presented with 95% confidence intervals (CIs). (p < 0.05.)

*Statistically significant (p < 0.05)

### Outcomes

The primary outcome was a 30-day composite postoperative complication, defined as the occurrence of hematoma requiring surgical reoperation, wound dehiscence, or surgical site infection. Hematoma was defined as postoperative bleeding requiring surgical reoperation. Surgical site infection was defined clinically as abscess or cellulitis documented by the treating physician, and wound dehiscence as wound separation documented in the medical record. Only complications documented in the available medical records were included in the analysis. Secondary outcomes included the individual complication components, drain duration, and length of hospital stay. Outcome data were extracted from electronic medical records by a single reviewer using the predefined outcome definitions described above.

### Statistical Analysis

All statistical analyses were performed using SPSS version 27 (IBM Corp., Armonk, NY, USA). Continuous variables were summarized as mean ± standard deviation or median (range), as appropriate, and categorical variables as frequencies and percentages. Normality was assessed using the Shapiro–Wilk test together with graphical inspection. Between-group comparisons were performed using Student’s t-test or one-way analysis of variance for normally distributed variables, and Mann–Whitney U or Kruskal–Wallis tests for non-normally distributed variables.

Categorical variables were analyzed using Pearson’s chi-square test, Fisher’s exact test, or Fisher–Freeman–Halton test, as appropriate. All tests were two-sided, and p < 0.05 was considered statistically significant. To evaluate factors associated with the primary outcome, a multivariable logistic regression model with backward stepwise selection was constructed. The dependent variable was composite postoperative complication (0 = no, 1 = yes), and block administration (yes vs no) was entered as the main exposure variable. Clinically relevant covariates, including age, body mass index, total excision weight, and nipple transposition distance, were entered into the initial model.

Exploratory subgroup analyses were performed to compare outcomes across block subtypes using appropriate statistical tests for categorical variables.

Adjusted odds ratios with 95% confidence intervals and p-values were reported. Multicollinearity among covariates was assessed before model construction. Given the non-randomized design, possible confounding by indication was taken into account in the interpretation of the findings.

A post-hoc power analysis was performed for the primary composite outcome comparison using the observed complication rates in the block and no-block groups. The effect size was calculated using Cohen’s h for two independent proportions. Power was calculated with a two-sided alpha level of 0.05 using the observed group sizes. The total sample size required to achieve 80% power for the observed effect size was also estimated while preserving the approximate observed allocation ratio. The post-hoc power analysis was performed using G*Power version 3.1.9.7.

## Results

### Patient Characteristics

A total of 144 female patients underwent bilateral superior pedicle reduction mammaplasty. The mean age was 38.6 ± 12.6 years (range, 16–68 years), and the mean body mass index (BMI) was 28.2 ± 3.9 kg/m^2^.

Of these, 64 patients (44.4%) received a regional fascial plane block (block group), whereas 80 (55.6%) did not (no-block group). Among patients in the block group, 15 (10.4%) underwent a SAPB, 33 (22.9%) a PECS II block, and 16 (11.1%) an SPSIP block.

Age and BMI were comparable between groups (p=0.626 and p=0.839, respectively).

The mean right-sided excision weight was 672.1 ± 332.9g in the block group compared with 827.1 ± 340.4g in the no-block group (p=0.007), and the mean left-sided excision weight was 659.0 ± 338.4g versus 797.3 ± 335.4g, respectively (p=0.015). The mean total excision weight across the cohort was 1489.4 ± 670.5g.

Preoperative right sternal notch-to-nipple (SNN) distance was lower in the block group (29.44 ± 2.64 cm vs. 30.65 ± 3.71 cm, p=0.031), whereas left-sided preoperative SNN measurements were similar between groups (p=0.139). Postoperative SNN distance was slightly higher in the block group (20.18 ± 0.59 cm vs 19.64 ± 0.88 cm, p=0.001) (Table 1).

### Primary Outcome: Composite Early Postoperative Complications

Overall, 55 of 144 patients (38.2%) experienced at least one early postoperative complication.

The composite complication rate was higher in the block group compared with the no-block group (46.9% [30/64] vs. 31.3% [25/80]), although this difference did not reach statistical significance (p=0.060) (Fig. 1; Table 2).

In multivariable logistic regression analysis, total excision weight emerged as an independent predictor of complications (OR 1.001, 95% CI 1.000–1.002; p=0.016). Block use was associated with higher odds of complications; however, this association did not reach statistical significance (OR 2.45, 95% CI 0.97–6.17; p=0.058). Age, BMI, and nipple transposition distance were not significantly associated with complications (Table 3).

Post-hoc power analysis for the primary composite outcome comparison showed that the observed difference in composite complication rates corresponded to a Cohen’s h of 0.322. With the current group sizes and a two-sided alpha level of 0.05, the achieved power was 48.4%. To detect the same effect size with 80% power, an estimated total sample size of approximately 307 patients would be required. Under the observed allocation ratio, this would correspond to approximately 136 patients in the block group and 171 patients in the no-block group.

### Block Subtype Analysis

In an exploratory subgroup analysis, composite complication rates varied across block subtypes (p=0.019). The rates were 31.3% (25/80) in the no-block group, 20.0% (3/15) in the serratus anterior group, 57.6% (19/33) in the PECS II group, and 50.0% (8/16) in the SPSIP group.

### Secondary Outcomes

#### Hematoma

A total of five hematomas (3.5%) were observed in the cohort. All events occurred in the block group (5/64 [7.8%]), whereas no hematomas were observed in the no-block group (0/80 [0%]; p=0.016) (Fig. 2).

When examined by block subtype, hematoma rates also differed (p=0.023), with rates of 6.7% (1/15) in the serratus anterior group, 9.1% (3/33) in the PECS II group, and 6.3% (1/16) in the SPSIP group. Given the small number of events (n=5), no multivariable analysis was performed for this outcome.

#### Wound Dehiscence

Wound dehiscence was observed in 50 patients (34.7%). The rate was higher in the block group compared with the no-block group (40.6% vs. 30.0%), although this difference was not statistically significant (p=0.183).

In the subgroup analysis, rates varied across block types (p=0.042), with higher rates in the PECS II and SPSIP groups compared with the serratus anterior group.

#### Infection

Infection occurred in 4 patients (2.8%), with similar rates between the no-block and block groups (3/80 [3.8%] vs. 1/64 [1.6%], p=0.629).

#### Drain Duration and Length of Hospital Stay

Mean drain duration was comparable between groups (4.05 ± 1.24 days in the no-block group vs 3.97 ± 1.47 days in the block group, p=0.516). Similarly, the mean length of hospital stay was nearly identical (4.16 ± 1.21 vs. 4.17 ± 1.22 days, p=0.975).

## Discussion

In this retrospective cohort, patients who received a regional fascial plane block initially appeared to have higher complication rates; however, this difference was no longer present after adjustment. Instead, total excision weight emerged as the only independent predictor. Given that excision weight is measured as a continuous variable, even small incremental increases may accumulate to a clinically relevant difference across larger resections. Differences of several hundred grams—commonly encountered in clinical practice—may therefore be relevant in terms of complication risk. This suggests that complications are more closely related to the magnitude of surgery than to block use itself. Similar observations have been reported in the reduction mammaplasty literature, where increasing resection weight has been associated with higher complication rates [16]. Large database studies have also shown that patient-related factors such as body mass index and smoking are associated with increased complication risk [17]. Taken together, these findings suggest that surgical burden may be more relevant in determining complications than the use of regional blocks. Information on postoperative complication profiles of regional fascial plane blocks in reduction mammaplasty remains limited, as most of the existing literature has focused on analgesic outcomes and provides little detail on surgical complications [18, 19].

Reported rates of hematoma requiring reoperation after reduction mammaplasty are low, around 1.8% in contemporary series [20]. In our cohort, hematoma was seen only in the block group (7.8% vs. 0%), and this difference was significant in univariate analysis. This is notable, particularly because excision weights were actually lower in the block group. However, the number of hematoma events was small (n=5), so this variable could not be included in the multivariable model. For this reason, an independent association cannot be confirmed.

Overall, block use did not seem to result in a worse postoperative course. Infection rates, drain duration, and length of stay were similar between groups, and most events in the composite outcome were minor wound dehiscence cases managed conservatively. Although the crude analysis showed higher complication rates in the block group, this difference was not observed after adjustment. Given the baseline differences between groups, this is likely related to confounding rather than a true effect of block use.

The timing and composition of the block may be relevant when interpreting the hematoma finding. In our series, all blocks were performed postoperatively using 30 mL of 0.25% bupivacaine without epinephrine, in line with routine institutional practice. The absence of epinephrine may be relevant because a vasoconstrictive effect at the injection plane would not be expected. However, local anesthetic composition varies across published regional block protocols, and the available data do not allow us to determine whether block timing or the absence of epinephrine contributed to hematoma formation [8, 21]. Therefore, the hematoma finding should be interpreted cautiously and not as evidence of a direct block-related effect.

When the analysis was broken down by block subtype, higher complication rates were seen in the PECS II and SPSIP groups. The number of patients in each subgroup was small, so this finding should be interpreted cautiously. In reduction mammaplasty, studies on regional blocks have mostly focused on analgesic outcomes [22, 23], and complication data are either limited or not reported in detail [24]. Direct comparison with the existing literature is therefore difficult, and these subgroup findings should be interpreted cautiously given the limited sample size.

The baseline differences between groups should be considered when interpreting these findings. Excision weights were lower in the block group, and some preoperative measurements also differed between groups. Although these variables were adjusted for in the regression model, adjustment can only account for measured factors. Since block selection was not based on a predefined protocol, residual selection bias cannot be excluded when interpreting both the main block versus no-block comparison and the exploratory block-subtype analyses.

Overall, our findings add complication-related data to the use of regional fascial plane blocks in reduction mammaplasty. In this cohort, block use was not associated with increased postoperative morbidity after adjustment; however, the wide confidence interval indicates that a relevant increase in complication risk cannot be ruled out. Therefore, these findings should be interpreted cautiously and not as definitive evidence of safety. The post-hoc power analysis further supports a cautious interpretation of the primary outcome. The achieved power was limited for the observed difference in composite complication rates, indicating that this comparison was not adequately powered to exclude a relevant difference between groups. Previous studies in reduction mammaplasty have mainly evaluated pain outcomes and have consistently shown reductions in early postoperative pain and opioid consumption [25], while data on surgical complications remain limited. In this respect, our study contributes additional data on complication profiles. Furthermore, all procedures were performed by a single surgeon using a consistent technique, reducing procedural variability and enhancing the internal validity of the findings.

This study has several limitations. Its retrospective design and non-standardized block allocation introduce a risk of selection bias. The small number of hematoma events limits the interpretation of this endpoint. Operative time, intraoperative variables, and detailed block parameters were not consistently available. Detailed data on drain output patterns, timing of hematoma diagnosis, and reoperation details were not available, limiting our ability to further explore the hematoma finding. Postoperative events managed at outside institutions may not have been captured in our records, which could have led to underreporting of complications. Outcome ascertainment relied on retrospective review of routine clinical documentation by a single reviewer; therefore, inter-rater reliability could not be assessed. Finally, only short-term postoperative outcomes were evaluated. These findings therefore reflect a single-center experience and may limit the generalisability of the results. Residual confounding by indication cannot be excluded despite adjustment.

## Conclusion

In this retrospective cohort, the use of regional fascial plane blocks in reduction mammaplasty was not associated with increased overall postoperative morbidity after adjustment for confounders. Total excision weight emerged as the main determinant of complication risk. However, the wide confidence interval for block use indicates that a relevant increase in complication risk cannot be ruled out. Although hematomas were observed only in the block group, the small number of events and the non-randomized design preclude definitive conclusions. These findings should be interpreted cautiously, particularly with regard to bleeding-related outcomes. Further prospective studies are needed to better define the relationship between block techniques, timing, local anesthetic composition, and bleeding-related outcomes in reduction mammaplasty.
